# The systems biology simulation core library

**DOI:** 10.1093/bioinformatics/btab669

**Published:** 2021-09-23

**Authors:** Hemil Panchiwala, Shalin Shah, Hannes Planatscher, Mykola Zakharchuk, Matthias König, Andreas Dräger

**Affiliations:** Department of Computer Science and Engineering, Indian Institute of Technology, Roorkee, IN 247667, USA; Department of Electrical and Computer Engineering, Duke University, Durham, NC 27701, USA; Bloomberg, New York, NY 10022, USA; Signatope GmbH, Reutlingen 72770, Germany; Department of Computer Science, University of Tübingen, Tübingen 72076, Germany; Institute for Theoretical Biology, Humboldt University of Berlin, Berlin 10115, Germany; Department of Computer Science, University of Tübingen, Tübingen 72076, Germany; Computational Systems Biology of Infections and Antimicrobial-Resistant Pathogens, Institute for Bioinformatics and Medical Informatics (IBMI), University of Tübingen, Tübingen 72076, Germany; German Center for Infection Research (DZIF), Partner Site Tübingen, Tübingen 72076, Germany; Cluster of Excellence ‘Controlling Microbes to Fight Infections,’ University of Tübingen, Tübingen 72076, Germany

## Abstract

**Summary:**

Studying biological systems generally relies on computational modeling and simulation, e.g., model-driven discovery and hypothesis testing. Progress in standardization efforts led to the development of interrelated file formats to exchange and reuse models in systems biology, such as SBML, the Simulation Experiment Description Markup Language (SED-ML) or the Open Modeling EXchange format. Conducting simulation experiments based on these formats requires efficient and reusable implementations to make them accessible to the broader scientific community and to ensure the reproducibility of the results. The Systems Biology Simulation Core Library (SBSCL) provides interpreters and solvers for these standards as a versatile open-source API in Java^TM^. The library simulates even complex bio-models and supports deterministic Ordinary Differential Equations; Stochastic Differential Equations; constraint-based analyses; recent SBML and SED-ML versions; exchange of results, and visualization of *in silico* experiments; open modeling exchange formats (COMBINE archives); hierarchically structured models; and compatibility with standard testing systems, including the Systems Biology Test Suite and published models from the BioModels and BiGG databases.

**Availability and implementation:**

SBSCL is freely available at https://draeger-lab.github.io/SBSCL/ and via Maven Central.

**Supplementary information:**

Supplementary data are available at *Bioinformatics* online.

## 1 Introduction

**Fig. 1.**
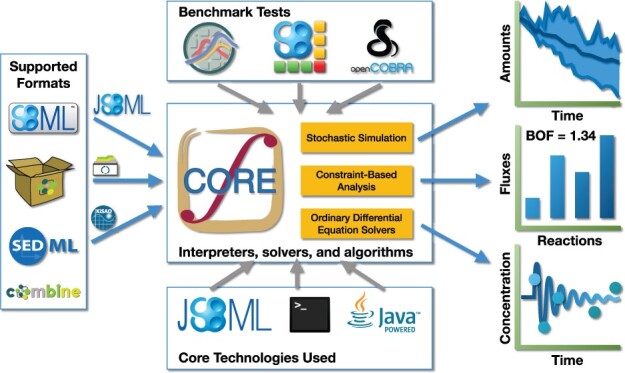
The capabilities of the SBSCL as an overview. Supported input model definitions include SBML, possibly with an experiment configuration file (SED-ML) or bundled in a package file (OMEX). The model is parsed using the JSBML library, and solutions are numerically computed for the corresponding ODE or SDE system over time, following the specified constraints and algorithm (e.g., Rosenbrock, Euler, Gillespie) or via linear programming. Once the simulation completes, the model results are reported either graphically using a line plot or tabular form. The results can be exported to formats such as CSV for downstream use. For testing the library, its implementation, its robustness, reliability, and efficient reproducibility of the results, open model collections such as BiGG (Norsigian *et al.*, 2019) and BioModels (Malik-Sheriff *et al.*, 2020) are utilized, which comprise several hundred SBML models and their SED-ML configurations

The Systems Biology Simulation Core Library (SBSCL) is an open-source, cross-platform pure Java^TM^ programming library that numerically solves systems biology models in multiple mathematical frameworks. A popular file format for representing computational models in a standard way and facilitating the exchange of models between different tools is the Systems Biology Markup Language (SBML, Keating *et al.*, 2020). SBML encodes biological models in a declarative form. The Simulation Experiment Description Markup Language (SED-ML) format defines a workflow of simulation experiments. The combination of SED-ML and SBML facilitates reproducibility of typical model workflows in *in* *silico* experiments, including the choice of interpretation framework and the post-processing of the results (Waltemath *et al.*, 2011). SBSCL interprets the SBML models using the JSBML library (Rodriguez *et al.*, 2015) and simulates them according to dedicated API calls. Alternatively, it extracts an *in silico* experimental configuration from SED-ML to simulate the SBML models. To this end, SBSCL implements and ships several solvers for a wide range of mathematical frameworks, including Ordinary Differential Equations (ODEs, Keller *et al.*, 2013), Stochastic Differential Equations (SDEs, Erhard *et al.*, 2008) and constraint-based analysis. SBSCL is designed as a lightweight API and intended for use as a simulation backend within end-user software. This article introduces the SBSCL library, especially the new features introduced in version 2.1, along with a brief description of all other capabilities that Figure 1 pictorially summarizes.

## 2 Description

*Differential equation solver*: The most fundamental feature of SBSCL is simulating ODEs. Version 2.1 adds interpreters and solvers for SDEs to support the latest SBML standards. SBSCL efficiently implements three deterministic numerical solvers (Keller *et al.*, 2013), namely, Rosenbrock, Euler and Runge-Kutta, as well as three stochastic solvers, namely, Gillespie, Gibson-Bruck and Tau-Leaping (Erhard *et al.*, 2008).

*Constrained optimization solver*: SBML Level 3 (Keating *et al.*, 2020) combined with the fbc package added support for constrained-based models and their analysis. Typically, Flux Balance Analysis (FBA) is used for such time-invariant steady-state simulations. SBSCL performs FBA on SBML models using the SCPSolver (http://www.scpsolver.org), a linear programming API with support for various solver backends. This lightweight abstraction allows users to define model constraints and an objective function and solve the corresponding optimization problem.

*Result tables and plots*: Since viewing is an essential aspect of understanding the results of a simulation experiment, SBSCL provides experiment output in graphical and tabular form, which it can export in conventional formats such as Comma-Separated Values (CSV).

*Archival format support*: Working toward exchangeability and reproducibility, SBSCL v2.1 supports the Open Modeling EXchange format (OMEX) format as input. These archive files contain the information on running simulation experiments based on SBML and SED-ML (Bergmann *et al.*, 2014). SBSCL uses the COMBINE Archive Simulation Experiment Management for Systems Biology (SEMS) package to read and extract the required information from the OMEX files.

*Hierarchical model simulations*: The SBML extension package comp enables encoding complex and coupled biological systems that can be distributed or hierarchically structured. SBSCL v2.1 efficiently supports the simulation of this addition, including the automatic assembly of models from multiple and possibly remote input files.

*Tests against benchmark suites*: A crucial part of implementing new features is providing robust testing of the added functionality and use-cases. SBSCL tests all newly added features against the SBML Test Suite in a continuous integration approach. SBSCL provides full testing support against the genome-scale models from the BiGG Models database and kinetic models from the BioModels database.

## 3 Conclusion

The open-source library SBSCL simulates complex biological models in various frameworks specified in SBML format, optionally together with their *in silico* experiment definition SED-ML file or wrapped within OMEX archives. Benchmarks of SBSCL using the SBML Test Suite and a broad range of published models from relevant databases ensure its correctness and reliability (see Supplementary Material). With the support for exciting new features such as constraint-based model optimization, hierarchical model decomposition, stochastic algorithms, archival input formats, this lightweight library is well suited as a simulation engine within any software with support for the Java Virtual Machine, e.g., Kotlin, Scala or Groovy. The SBSCL project aims to provide a high-quality open-source simulation library to the scientific community to push frontiers and reproducibility in biology and related fields.

## Supplementary Material

btab669_Supplementary_Data
