# Supplementary material for: The systems biology simulation core library
Source: Bioinformatics. 2021 Sep 23;38(3):864–5. doi: 10.1093/bioinformatics/btab669 (PMC8756180; doi:10.1093/bioinformatics/btab669)
Supplement: btab669_Supplementary_Data [file btab669_supplementary_data.pdf]

Supplementary Material

The Systems Biology Simulation Core Library

Hemil Panchiwala[1†], Shalin Shah[2,3†], Hannes Planatscher[4], Mykola Zakharchuk[5], Matthias König[6], and Andreas Dräger[5,7,8,9,*]

[1]Department of Computer Science and Engineering, Indian Institute of Technology, Roorkee, India - 247667
[2]Department of Electrical and Computer Engineering, Duke University, Durham, NC 27701, United States of America
[3]Bloomberg LP, New York, NY 10022, United States of America
[4]Signatope GmbH, 72770 Reutlingen, Germany
[5]Department of Computer Science, University of Tübingen, 72076 Tübingen, Germany
[6]Institute for Theoretical Biology, Humboldt University of Berlin, 10115 Berlin, Germany
[7]Computational Systems Biology of Infections and Antimicrobial-Resistant Pathogens, Institute for Bioinformatics and Medical Informatics (IBMI), University of Tübingen, 72076 Tübingen, Germany
[8]German Center for Infection Research (DZIF), partner site Tübingen, Germany
[9]Cluster of Excellence 'Controlling Microbes to Fight Infections,' University of Tübingen, Tübingen, Germany
[†]These authors contributed equally to the manuscript.
[*]Corresponding author: draeger@informatik.uni-tuebingen.de

ABSTRACT

Summary: Studying biological systems generally relies on computational modelling and simulation, e.g., model-driven discovery and hypothesis testing. Progress in standardisation efforts led to the development of interrelated file formats to exchange and reuse models in systems biology, such as SBML, the Simulation Experiment Description Markup Language (SED-ML), or the Open Modeling EXchange format (OMEX). Conducting simulation experiments based on these formats requires efficient and reusable implementations to make them accessible to the broader scientific community and to ensure the reproducibility of the results. The Systems Biology Simulation Core Library (SBSCL) provides interpreters and solvers for these standards as a versatile open-source API in Java™. The library simulates even complex bio-models and supports deterministic Ordinary Differential Equations (ODEs); Stochastic Differential Equations (SDEs); constraint-based analyses; recent SBML and SED-ML versions; exchange of results, and visualisation of *in silico* experiments; open modelling exchange formats (COMBINE archives); hierarchically structured models; and compatibility with standard testing systems, including the Systems Biology Test Suite and published models from the BioModels and BiGG databases.
Availability: SBSCL is freely available at https://draeger-lab.github.io/SBSCL/ and via Maven Central.

Keywords: Systems Biology, Numerical Solver, Java™, API Library, SBML, SED-ML, OMEX, Constraint-Based Modelling, Stochastic Simulation, Ordinary Differential Equation Systems

Contents

1 Resources and availability **2**
1.1 Installation via Maven . . . 2
1.2 Use-case examples . . . 2
Fundamentals for working with SBML in Java • Deterministic dynamic simulation • Stochastic simulation • Constraint-based analysis • Simulating a hierarchically structured model • Simulating SED-ML documents • Simulation of OMEX files
2 Software design and implementation **7**
3 Benchmark tests **7**
3.1 Support of the SBML Test Suite . . . 8
3.2 BioModel simulations . . . 10
3.3 BiGG Model simulations . . . 10
3.4 Comparison to other simulators with SBML support . . . 18
AMICI • BioUML • COPASI • FluxBalance • iBioSim • LibRoadRunner • LibSBMLSim • ModelBase • Morpheus • WinBEST-KIT
4 Known limitations **20**
References **21**

1 Resources and availability

SBSCL is a freely available open-source library for analysis, simulation, and interpretation of systems biology models in various modelling frameworks.

- The primary repository of SBSCL is available at /draeger-lab/SBSCL.
- A demo repository is available at /draeger-lab/SBSCL-demo.
- The project /matthiaskoenig/sbscl-simulator-comparison provides benchmarks of SBSCL against other simulators.

1.1 Installation via Maven

The repository of SBSCL is based on Maven. Using SBSCL is straightforward when adding it as a dependency to the Project Object Model (POM) file in a Java™ project, as listing 1 demonstrates. This declaration automatically loads also all transitive dependencies, i.e., all required third-party libraries.

The version number will increase when new releases of SBSCL become available and will be listed in the README.md file of the primary repository. A minimal example, demonstrating the use and how a simple POM file could be structured, is available within the demo repository that is ready to use and try out. The standalone application SBMLsimulator[6,8] provides a Graphical User Interface (GUI) for SBSCL and is freely available.

For illustration purposes, listing 2 below also shows a template for such a minimal pom.xml file. All that needs to be changed to make this minimal example work is filling in values for the placeholders [YOUR GROUP ID], [YOUR ARTIFACT ID], [A MEANINGFUL NAME], [VERSION NUMBER OF YOUR PROJECT].

Listing 1. Declaring SBSCL as a dependency within a Maven POM file

```
<dependency>
  <groupId>org.draegerlab</groupId>
  <artifactId>sbscl</artifactId>
  <version>2.1</version>
</dependency>
```

1.2 Use-case examples

We will now discuss a few use-case examples to demonstrate using SBSCL as a solver engine within an application. Just like the example POM file above, the examples described in this section can also be found in the demo repository for SBSCL at /draeger-lab/SBSCL-demo in the form of fully functioning standalone programs. The online JavaDoc provides further information about the details and functioning of the classes below. Trying out the example programs below requires downloading models in Systems Biology Markup Language (SBML) format from one of the preeminent online databases, such as BioModels[27] or BiGG[24,30] . For more general advice in developing larger software projects, we refer to related publications[47].

1.2.1 Fundamentals for working with SBML in Java

To reduce the examples to the main aspects relevant to working with SBSCL directly, we will first discuss a few general techniques so that the following source-code examples can build upon these. The JSBML library[10,34] efficiently parses SBML files and delivers an object of type `SBMLDocument`, as listing 3 on the following page demonstrates. In most following examples, we will assume that the `main` method is present and that it will just call the constructor of the example class by passing an `SBMLDocument` object to it as an argument. By this, we can assume the user needs to specify the path to the SBML file of interest as a command-line argument following the scheme outlined in listing 4 on the next page. The code below launches a demo application from the command line with the absolute or relative path to an SBML file as the only argument (`args[0]`).

All examples assume the given SBML file to include the necessary extension packages expected for the desired analysis. In practice, further case distinctions may be necessary to ensure that correct content is loaded. The SBML file will be parsed via JSBML[10,34], and the resulting `SBMLDocument` will be passed to the constructor of the class for further processing. Any exception will stop the program and automatically print a stack trace, e.g., if the file cannot be found, parsed, or is invalid. Finally, we recommend to always initialise a logger for the class to handle output, e.g., warnings, user messages, or more fine-grained debug information. This method is valuable because the output can be forwarded to the console, a log file, or some GUI. In our examples, we use the logger shipped within the standard distribution. Other third-party packages may provide more advanced features. The subsequent examples assume a logger to be bound to the variable `logger`, as demonstrated in listing 5 on the following page. We here call the containing class `MyClass` for demonstration purposes.

Listing 2. Example for a minimal Maven POM file

```xml
<?xml version="1.0" encoding="UTF-8"?>
<project xmlns="http://maven.apache.org/POM/4.0.0" xmlns:xsi="http://www.w3.org/2001/XMLSchema-instance" xsi:schemaLocation="http://maven.apache.org/POM/4.0.0 http://maven.apache.org/xsd/maven-4.0.0.xsd">

  <modelVersion>4.0.0</modelVersion>

  <!-- ================================================================== -->
  <!-- General project information                                         -->
  <!-- ================================================================== -->

  <groupId>[YOUR GROUP ID]</groupId>
  <artifactId>[YOUR ARTIFACT ID]</artifactId>
  <name>[A MEANINGFUL NAME]</name>
  <version>[VERSION NUMBER OF YOUR PROJECT]</version>

  <packaging>jar</packaging> <!-- Output to jar format -->

  <properties>
    <jdk.version>1.8</jdk.version>
    <maven.build.timestamp.format>yyyy</maven.build.timestamp.format>
    <year>${maven.build.timestamp}</year>
    <project.build.sourceEncoding>UTF-8</project.build.sourceEncoding>
  </properties>

  <!-- ================================================================== -->
  <!-- Dependencies                                                        -->
  <!-- ================================================================== -->

  <dependencies>
    <dependency>
      <groupId>org.draegerlab</groupId>
      <artifactId>sbscl</artifactId>
      <version>2.1</version>
    </dependency>
  </dependencies>

  <!-- ================================================================== -->
  <!-- Building                                                            -->
  <!-- ================================================================== -->

  <build>
    <pluginManagement>
      <plugins>
        <plugin>
          <groupId>org.apache.maven.plugins</groupId>
          <artifactId>maven-compiler-plugin</artifactId>
          <version>3.3</version>
          <configuration>
            <source>${jdk.version}</source>
            <target>${jdk.version}</target>
          </configuration>
        </plugin>
      </plugins>
    </pluginManagement>
  </build>

</project>
```

Listing 3. Parsing an SBML file with JSBML

```java
SBMLDocument doc = SBMLReader.read(new File("/path/to/my/sbml/file.xml"));
```

Listing 4. Example for launching the application `DemoConstructor` with an SBML file as a command line argument

```java
public static void main(String[] args) throws Exception {
  new DemoConstructor(SBMLReader.read(new File(args[0])));
}
```

Listing 5. Initialising a logger for a sample class here called `MyClass`

```java
private static final transient Logger logger = Logger.getLogger(MyClass.class.getName());
```

Listing 6. Static import of the `format` method in the header of a Java class

```java
import static java.text.MessageFormat.format;
```

Listing 7. Example for running a deterministic dynamic simulation

```java
public DynamicSimulationDemo(SBMLDocument doc, double timeEnd) throws Exception {
  SBMLinterpreter interpreter = new SBMLinterpreter(doc.getModel());
  AbstractDESSolver solver = new RosenbrockSolver();
  MultiTable solution = solver.solve(interpreter, interpreter.getInitialValues(), 0d, timeEnd);
  // Print, plot, or display the solution as a table...
}
```

It is also recommended to enable localisation support via the standard Java class `MessageFormat`, whose `format` method can be statically imported as listing 6 indicates. Once this is done, it can be directly called from anywhere within the Java class, just like any other method.

1.2.2 Deterministic dynamic simulation

Listing 7 demonstrates how to run a deterministic simulation by interpreting an SBML file as an ODE system. We assume the constructor to be called from the `main` method with two values: an `SBMLDocument` and a number that gives the end time for the simulation. SBML assumes simulations to always start at time $t = 0$, but SBSCL provides a more general implementation that allows the solver to work with other initial time points. It is also possible to pass an array with monotonously increasing `double` values to the solver to enforce that exact time points are met within the simulation. This technique can be crucial for model calibration, e.g., to compare the simulation's output to experimentally obtained values at specific time points. Many solvers are directly available within SBSCL (see figure 1 on page 7). We here use the `RosenbrockSolver` solver because it provides step-size adaptation and fine-grained error estimation, making it most precise and robust against stiff ODEs[23]. However, other solvers may have a faster runtime.

The result is a table data structure, which SBSCL calls `MultiTable`. This table possesses a shared time column for multiple data blocks. Such data blocks allow the simulation results to be organised in separate spreadsheets for compartments, reactive species, fluxes, and variable parameters. It is possible to access these values separately, display them as tables, write them to files, e.g., in Comma-Separated Values (CSV) format, or plot them directly using some suitable plotting framework.

The `solve` method of the `DESSolver` interface, from which the class `AbstractDESolver` inherits, also allows passing a customised observer as an additional argument. Such an observer needs to implement the `PropertyChangeListener` interface from the `java.beans` package. It can be handy for listening to interim results or or tracking the progress of the simulation, e.g., to display a progress bar or to plot values as they roll in. More detailed descriptions of the algorithms in this example can be found in a separate publication[23].

It may look surprising that the `SBMLinterpreter` is asked to deliver initial values for the system. Those might not be directly stored in the SBML model but might be a target for more complex calculations, e.g., by solving initial assignments or other more involved operations. Of course, it is possible to pass customised initial conditions to the solver as needed. The solver also provides several settings, for instance, to adjust the step size as needed.

1.2.3 Stochastic simulation

Listing 8 on the following page shows a minimal example for running a stochastic simulation. Since the stochastic simulation part of SBSCL originates from the Framework for Evaluation of Reaction Networks (FERN) library[13] that uses a custom internal network to represent the SBML model, the procedure for loading the model diverges slightly from the other examples in this section. In the background, however, it also uses JSBML[10,34] for parsing the file.

To get the simulation result displayed, we need to initialise an observer now and add it to the simulator, in this case, an enhanced Gillespie solver[16]. The observer also requires a `double` value for the interval (comparable to the step size in deterministic simulation), an integer duration value, and an array of the species to be observed. As a general reference, the class `Start` within the package `fern` provides a fully-featured set of the stochastic simulation capabilities of SBSCL. Example files are, for instance, located in `/src/main/resources/examples/` within the project's repository.

1.2.4 Constraint-based analysis

Conducting a Flux Balance Analysis (FBA) belongs to the fundamental tasks in constraint-based modelling. Listing 9 on the next page shows a minimal example of how this can be done. This example assumes the SBML file to be parsed before launching the constructor of the class, here called `FBCdemo`. Any output is passed to a logger and displayed using the `format`

Listing 8. Example for running a stochastic dynamic simulation

```
public static void main(String args[]) throws Exception {
  Network net = NetworkTools.loadNetwork(new File(args[0]));
  Simulator sim = new GillespieEnhanced(net);
  ((SBMLNetwork) net).registerEvents(sim);
  String[] species = NetworkTools.getSpeciesNames(sim.getNet(),
    NumberTools.getNumbersTo(sim.getNet().getNumSpecies() - 1));
  Observer observer = new AmountIntervalObserver(sim, 0.1d, 5, species);
  sim.addObserver(observer);
  sim.start(5d); // end time
  observer.setPrintWriter(new PrintWriter(System.out));
  observer.print();
}
```

Listing 9. Example for running a flux balance analysis

```
public FBCdemo(SBMLDocument doc) throws Exception {
  FluxBalanceAnalysis solver = new FluxBalanceAnalysis(doc);
  if (solver.solve()) {
    logger.info(format("Objective␣value:\t{0}", solver.getObjectiveValue()));
    logger.info(format("Fluxes:\t{0}", solver.getSolution()));
  } else {
    logger.warning(format("Solver␣returned␣null␣for␣model␣{0}.", doc.getModel().getName()));
  }
}
```

method from the standard Java class `MessageFormat`. All that needs to be done to conduct an FBA, is instantiating an object of type `FluxBalanceAnalysis` by passing an `SBMLDocument` to it. The objective value and the flux distribution can then be obtained from this instance. It is also possible to change the actual solver, which is handled by the underlying SCPSolver. The BiGG[24,30] Models Database provides many example models suitable for flux balance analysis with listing 9.

1.2.5 Simulating a hierarchically structured model

Listing 10 provides a code snippet that demonstrates how to simulate a model that includes the SBML extension package for the Hierarchical Model Composition (`comp`) extension. In this example, the result is displayed in the form of a table in a simple GUI. The path to the SBML file is here given as an argument within the variable `args[0]`. In the background, the `CompSimulator` class uses the so-called "flattening" routine implemented in the JSBML library. Flattening means that the hierarchically structured model is converted to a non-hierarchical model in memory, i.e., with a "flat" hierarchy of only one level. Afterwards, a regular solver can be applied to it. This example includes an uncomplicated display of the simulation results in a table on a dialogue window for illustration purposes. The SBML Test Suite[22] comprises some `comp` models, such as semantic test № 1128 in `01128-sbml-l3v1.xml`, to run the code in listing 10.

1.2.6 Simulating SED-ML documents

The format SED-ML[45] has been designed to define the stages of a model's typical life cycle in a structured way:

1. Formulation of the model's equations
2. Specification of all necessary numerical values, such as initial conditions and kinetic parameters or boundary values
3. Simulation of the model in a specified framework
4. Post-processing and analysis of the result, e.g., graphical display.

Listing 10. Simulation of a hierarchically structured SBML model with `comp` extension

```
double timeEnd = 100d, stepSize = 0.1d;
CompSimulator compSimulator = new CompSimulator(new File(args[0]));
MultiTable solution = compSimulator.solve(timeEnd, stepSize);
// Display simulation result to the user
JScrollPane resultDisplay = new JScrollPane(new JTable(solution));
resultDisplay.setPreferredSize(new Dimension(400, 400));
JOptionPane.showMessageDialog(null, resultDisplay, "Comp␣Results", JOptionPane.INFORMATION_MESSAGE);
```

Listing 11. Simulation of an SBML model by interpreting instructions from a SED-ML file

```java
public static void main(String[] args) throws Exception {
  File file = new File(args[0]);
  SedML sedml = Libsedml.readDocument(file).getSedMLModel();
  // We assume our SED-ML file to have just one output. We could either iterate or get the user
  // to decide which one to run if there were several.
  Output wanted = sedml.getOutputs().get(0);
  SedMLSBMLSimulatorExecutor exe = new SedMLSBMLSimulatorExecutor(sedml, wanted, file.getParent());
  // This gets the raw simulation results - one for each Task that was run.
  logger.info("Collecting tasks...");
  Map<AbstractTask, List<IRawSedmlSimulationResults>> res = exe.run();
  if ((res == null) || res.isEmpty() || !exe.isExecuted()) {
    logger.warning(format("Simulatation failed: {0}", exe.getFailureMessages().get(0)));
    return;
  }
  // Now process: In this case, there is no processing performed - we are displaying the raw results.
  logger.info(format("Outputs wanted: {0}", wanted.getId()));
  IProcessedSedMLSimulationResults prRes = exe.processSimulationResults(wanted, res);

  if (wanted.isPlot2d()) {
    Plot2D plots = (Plot2D) wanted;
    // Plot all processed results as per curve descriptions.
    PlotProcessedSedmlResults p = new PlotProcessedSedmlResults(prRes, plots.getListOfCurves(), plots.
        ↪ getElementName());
    p.pack();
    RefineryUtilities.centerFrameOnScreen(p);
    p.setVisible(true);
  }
}
```

The example in listing 11 shows how a SED-ML file can be loaded, interpreted, and executed using SBSCL's solvers. It makes extensive use of the execution framework from the jlibsedml library[1]. This framework performs boiler-plate code for operations such as post-processing of results and much more. This example is based on SED-ML Level 1 Version 2[3] elements, such as `RepeatedTasks` and `FunctionalRange`. The directory `/src/test/resources/sedml/` within the SBSCL project contains example SED-ML files to test the code in listing 11.

1.2.7 Simulation of OMEX files

The Computational Modeling of Biological Networks (COMBINE) archive file format OMEX bundles many diverse files with a manifest file explaining the content[2]. This archive file format is most suitable for distributing many standardised files within one single compressed file. Consequently, an OMEX file may contain both a SED-ML file and an SBML file and possibly additional information, such as shared metadata[29].

Listing 12 indicates how to load an OMEX file directly and to run it with SBSCL. For the sake of simplicity, we here assume that the given OMEX archive includes a SED-ML file, which does not always have to be the case. Consequently, further checks may be recommendable for ensuring the stability of end-user software. We also omit any demonstration for further processing of the individual simulation results but refer the reader to the examples above.

The file `/src/test/resources/omex/12859_2014_369_MOESM1_ESM.omex` within the SBSCL project repository can be used to test the code in listing 12.

Listing 12. Simulation of an SBML model within a COMBINE archive OMEX file

```java
OMEXArchive archive = new OMEXArchive(new File(args[0]));

if (archive.containsSBMLModel() && archive.containsSEDMLDescp()) {
  // Execute SED-ML file and run simulations.
  SEDMLDocument doc = Libsedml.readDocument(archive.getSEDMLDescription());
  SedML sedml = doc.getSedMLModel();

  Output wanted = sedml.getOutputs().get(0);
  SedMLSBMLSimulatorExecutor exe = new SedMLSBMLSimulatorExecutor(sedml, wanted, archive.
      ↪ getSEDMLDescription().getParentFile().getAbsolutePath());

  Map<AbstractTask, List<IRawSedmlSimulationResults>> res = exe.run();

  for (List<IRawSedmlSimulationResults> re_list : res.values()) {
    for (IRawSedmlSimulationResults re : re_list) {
      MultTableSEDMLWrapper wrapper = (MultTableSEDMLWrapper) re;
      // Process the result.
    }
  }
}
```

2 Software design and implementation

Figure 1 provides a simplified overview of the most central classes of SBSCL that are needed for the interpretation and simulation of systems biology models in different frameworks. Already the first release of SBSCL shipped the solvers for ODEs. For version 2, the SBML interpreter has been improved to support several new models from the SBML Test Suite[22]. Also, several problems were solved, including efficiency improvements and reduction of redundant source code.

Hierarchically structured models that rely on the `comp` extension package[37] for SBML are supported via the so-called flattening routine of the JSBML library[10,34]. The term “flattening” means that the model is first converted to a non-hierarchical, i.e., conventional, model in a pre-processing step, after which it can be directly solved using existing interpreters and solvers. By this, the support for models of this type strongly depends on the flattening algorithm within JSBML.

The FERN library[13] has been updated and incorporated into SBSCL to support stochastic simulation. It is now available as a separate repository (/draeger-lab/FERN) for direct use. However, the maintenance continues within SBSCL, in which its classes have been adjusted for improved compatibility between the different frameworks. In doing so, the FERN package that initially depended on libSBML[5] was also migrated to JSBML.

New is also the package for constraint-based analysis, which currently contains the class `FluxBalanceAnalysis` and a linear solver based on the GNU Linear Programming Kit (GLPK). Additional Constraint-Based Reconstruction and Analysis (COBRA) methods are planned to be added in future releases and access to further linear solvers, which are included through the abstraction layer called SCPSolver.

3 Benchmark tests

We will now discuss the performance and accuracy of SBSCL based on the SBML Test Suite collection of edge-case models and published models from the online databases BioModels[27] and BiGG[24,30] and compare SBSCL to related software.

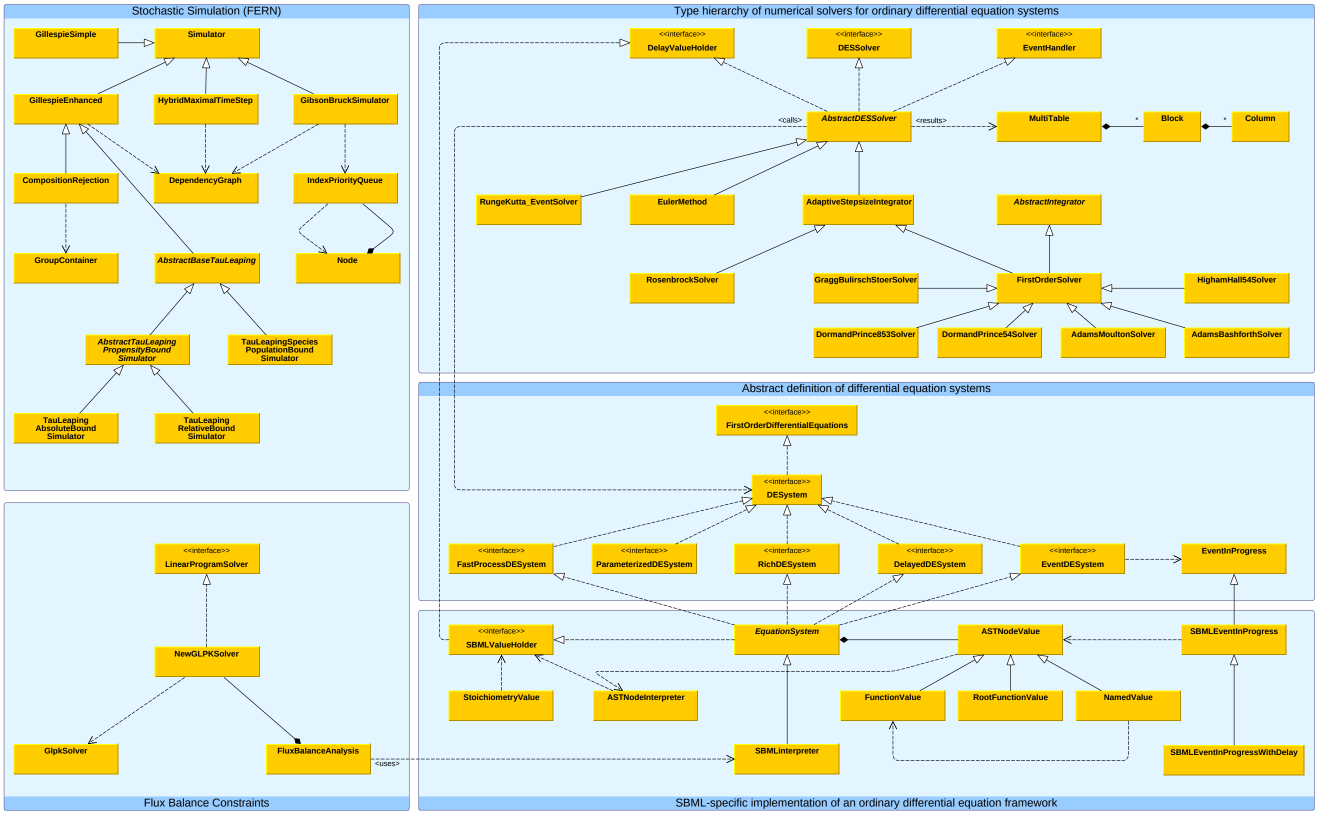

Figure 1. Overview of the type hierarchy of SBSCL (simplified). This Unified Modeling Language (UML) class diagram indicates that the library’s design separates numerical solvers from equation systems and separately defines interpreters of the systems biology model format SBML. This abstract design facilitates adding more formats, such as CellML[26], as soon as Java™ parsers become available. In addition, since version 2.0, SBSCL comes with two new packages: for “stochastic simulation” using the FERN library[13] and for “flux balance constraints” based on the SCPSolver project.

3.1 Support of the SBML Test Suite

When SBSCL version 1.2 was released, it passed all tests from the ever-expanding test suite[23]. Meanwhile, the number of tests for the SBML core has increased, as figure 2 shows. In addition, test cases for specific extension packages have been created to cover more aspects of SBML. Figure 3 on the next page shows that release 2.1 of SBSCL supports and correctly solves the vast majority of the test cases from the current SBML Test Suite for SBML core, including deterministic and stochastic simulation tests and all tests for constraint-based simulation. As soon as the so-called "flattening" algorithm for converting hierarchically structured SBML models into monolithic models in the underlying JSBML library[10,34] is completed, SBSCL will immediately support more test cases for this SBML extension package called `comp`[37].

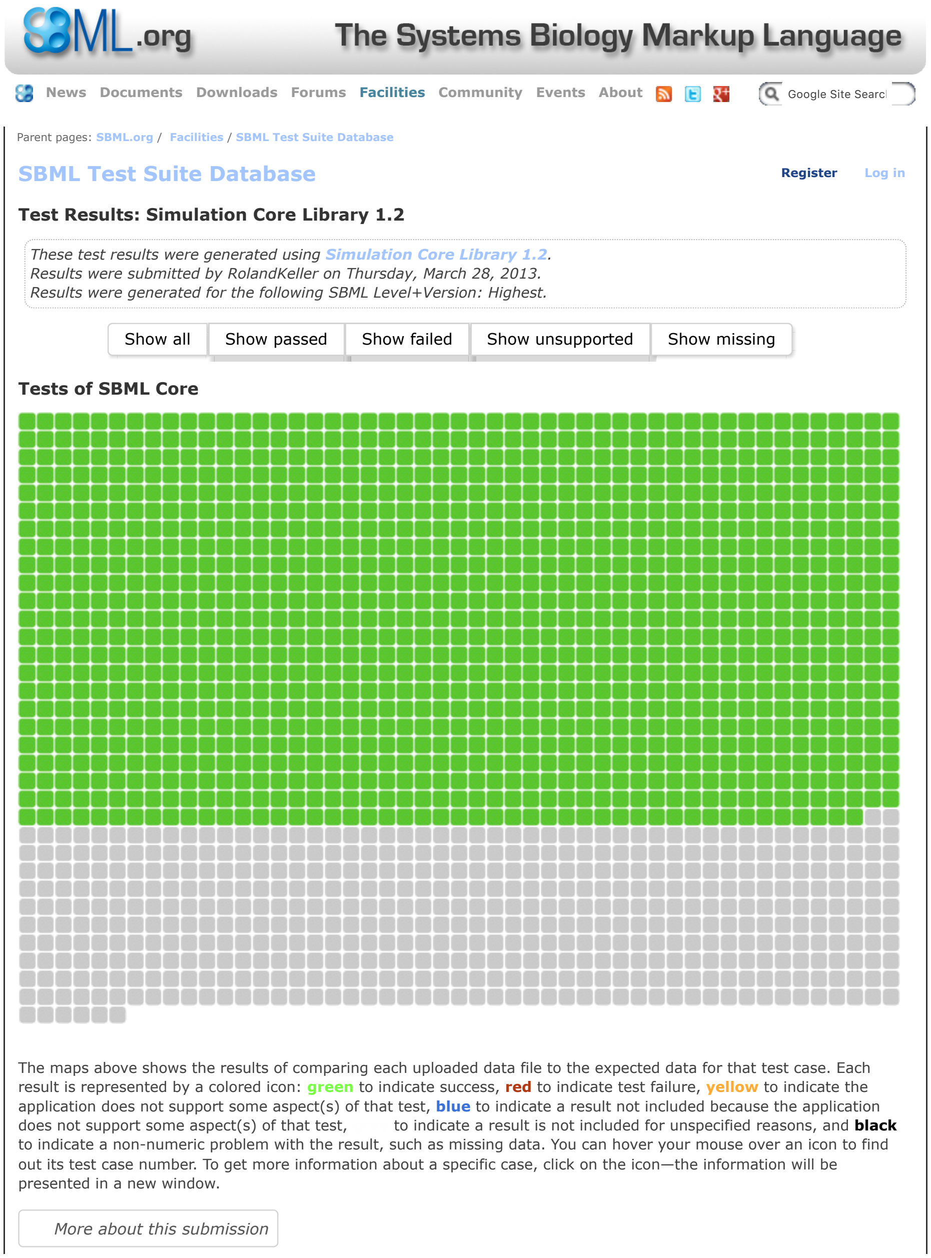

Figure 2. Test Results of the Systems Biology Simulation Core Library (SBSCL) version 1.2. This overview is accessible at `http://sbml.org/Facilities/Database/Submission/Details/67`.

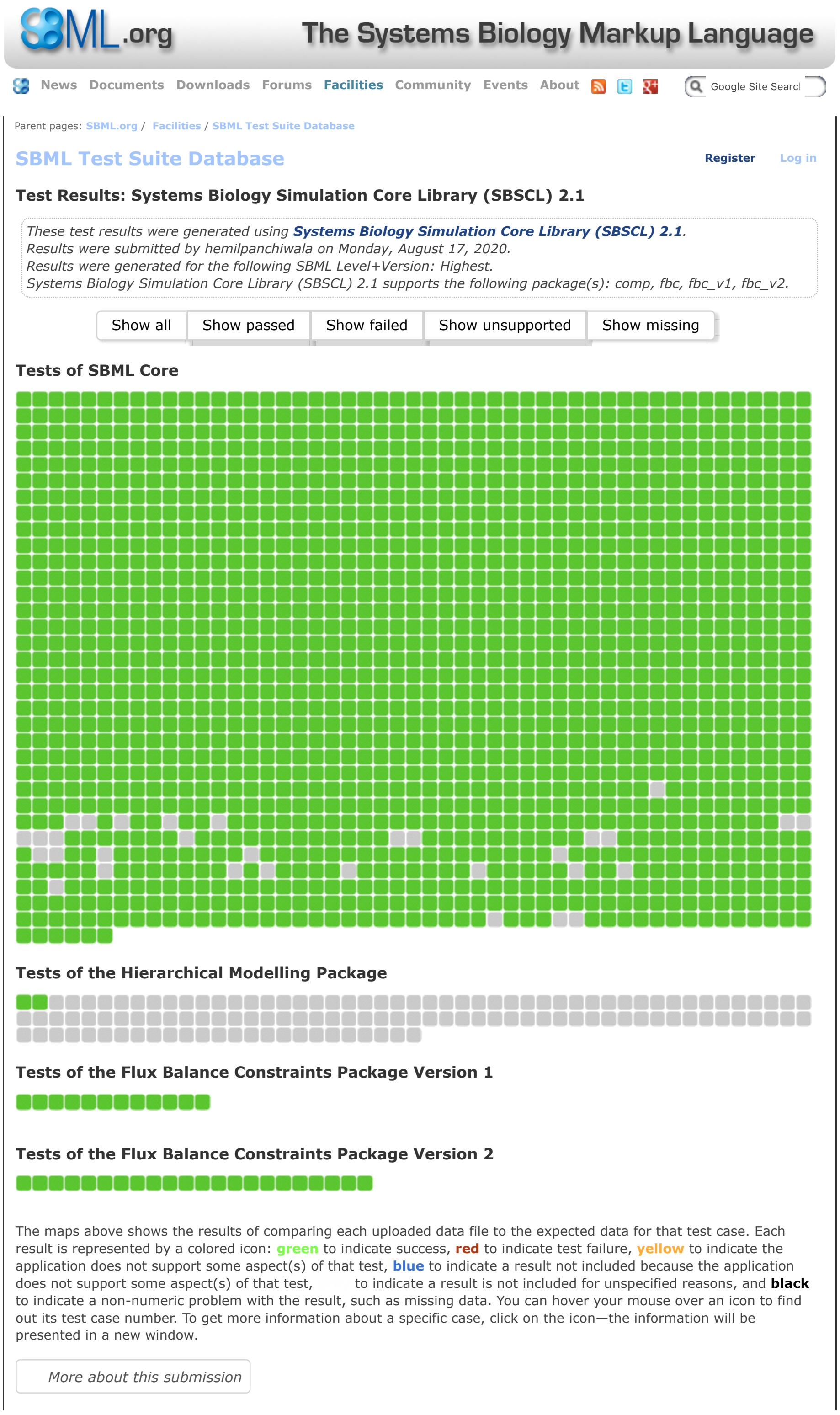

Figure 3. Test Results of the Systems Biology Simulation Core Library (SBSCL) version 2.1. This overview is available at http://sbml.org/Facilities/Database/Submission/Details/257.

3.2 BioModel simulations

The BioModels Database[27] is one of the most commonly used online repositories for curated models in various formats from the community for COMBINE[11]. The BioModels Database facilitates collaboration and promotes the reuse, sharing, and repurposing of computational models in systems biology[9].

To assess the usability and performance of SBSCL, we simulated the first 100 curated models from this online repository with SBSCL version 2.1, COmplex PAthway SImulator (COPASI) version 4.30.240[18], and RoadRunner version 2.0.5[40]. All simulations were executed on the same machine using a single core[1]. Model load time and simulation time were timed separately, and the total time was calculated as the sum of load and simulate time. Model loading and simulation were performed in five independent runs for every simulator. All settings were identically selected for all simulators and all models as follows:

- `start = 0`
- `stop = 100`
- `steps = 100`
- `absolute_tolerance = 1E-10`
- `relative_tolerance = 1E-6.`

for all models. SBSCL used the Rosenbrock solver with step-size adaptation. The comparison project provides the respective source code at /matthiaskoenig/sbscl-simulator-comparison.

Model load time is reported in figure 4 on the following page, simulation time in figure 5 on page 12, and total time in figure 6 on page 13. All 100 models could be executed successfully with SBSCL version 2.1, whereas COPASI version 4.30.240 and RoadRunner version 2.0.5 could execute 97 models successfully. However, due to the lacking support for delays in COPASI and RoadRunner, three models of circadian oscillations (BIOMD0000000024[35], BIOMD0000000025[38], BIOMD0000000034[39]) could not be executed by the respective simulators.

All models loaded substantially faster with SBSCL (see figure 4 on the next page), mainly due to additional just-in-time compilation steps required in COPASI and RoadRunner. Simulations were overall the fastest with RoadRunner, followed by COPASI and RoadRunner figure 5 on page 12. Considering the combined load and simulation time for a single simulation (figure 6 on page 13), SBSCL has a much broader spread than the other simulators. Some models execute faster or slower than others compared to COPASI version 4.30.240 and RoadRunner version 2.0.5. COPASI and RoadRunner have clear advantages when executing many simulations after loading the model once. SBSCL could show similar performance for many models for a single simulation due to the faster model loading times.

3.3 BiGG Model simulations

BiGG[24,30] Models is a genome-scale metabolic network reconstructions database that integrates more than 100 published genome-scale metabolic networks[30]. For benchmarking our software suite for the FBA models, we simulated the entire suite of BiGG[24,30] Models using SBSCL version 2.1 and compared it with results obtained from COBRApy version 0.21.0, a package for constraint-based modelling of metabolic networks based on the COBRA methods, which are widely used for genome-scale modelling of metabolic networks[12].

Model load time is reported in figure 7 on page 14, simulation time in figure 8 on page 15 and total time in figure 9 on page 16. All 100 models could be executed successfully with SBSCL version 2.1 and COBRApy version 0.21.0.

All models loaded substantially faster with SBSCL (see figure 7 on page 14) than with COBRApy. Simulations were drastically faster with COBRApy than with SBSCL (see figure 8 on page 15). The combined load and simulation time for a single simulation (figure 9 on page 16) modelling is faster than COBRApy. COBRApy has a clear advantage when executing many simulations after loading the model once, whereas, for a single simulation, SBSCL has advantages due to the faster model loading times.

The model simulations result in a predicted growth rate (in $\mathrm{mmol}/(\mathrm{gDW} \cdot \mathrm{h})$). Figure 11 on page 18 displays the overall divergence between the growth rate predictions of SBSCL and COBRApy, which is on the order of 10^{-8}. For most models, the difference approaches 0 as it is close to the numerical precision of the machine. Figure 10 on page 17 indicates the individual differences in growth rates for all models from BiGG[24,30].

[1]Hardware and software configuration: Acer Notebook of type Predator PH315-52 version 1.06 with an Intel® Core™ i7-9750H CPU at 2.60 GHz and 16 GB of main memory and Ubuntu Linux version 18.04.5 LTS as the operating system with OpenJDK version 1.8.0 232.

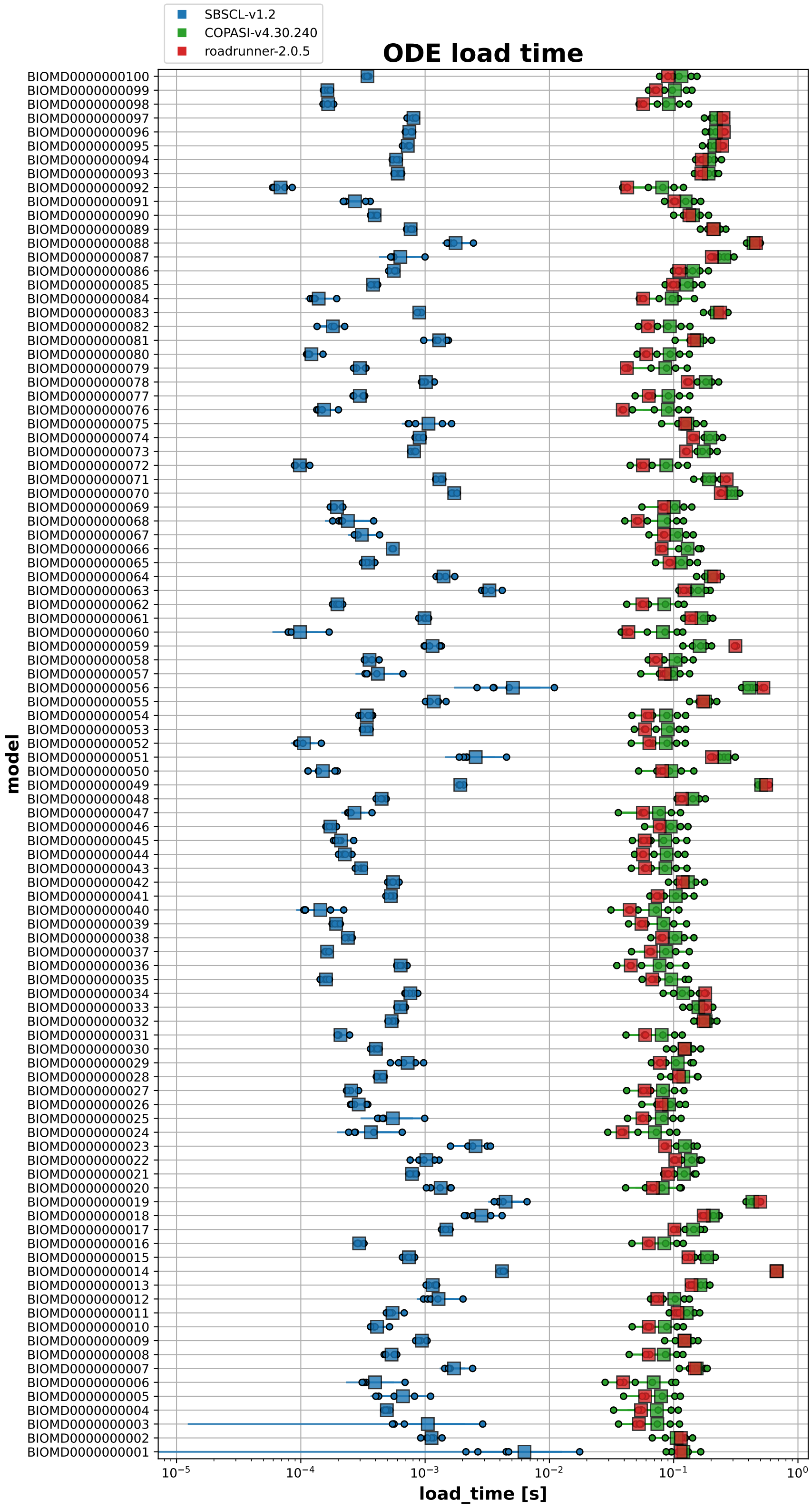

Figure 4. BioModels load time (in s). Displayed is mean and standard deviation for five repeats and the individual repeats for loading the first 100 models from BioModels Database[27] with SBSCL version 2.1, COPASI version 4.30.240[18], and RoadRunner version 2.0.5[40].

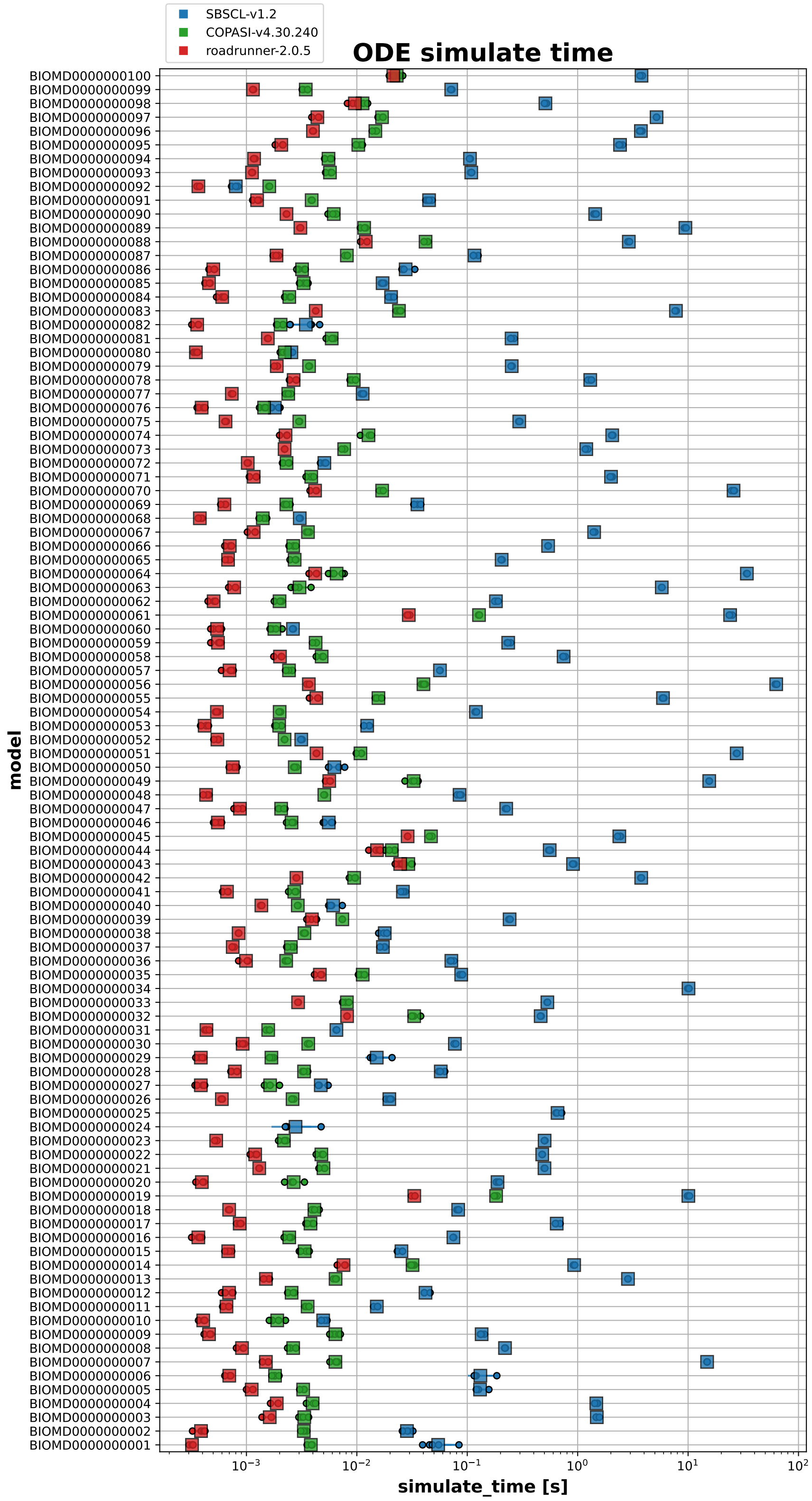

Figure 5. BioModels simulate time (in s). Displayed is mean and standard deviation for five repeats and the individual repeats for simulating the first 100 models from BioModels Database[27] with SBSCL version 2.1, COPASI version 4.30.240[18], and RoadRunner version 2.0.5[40].

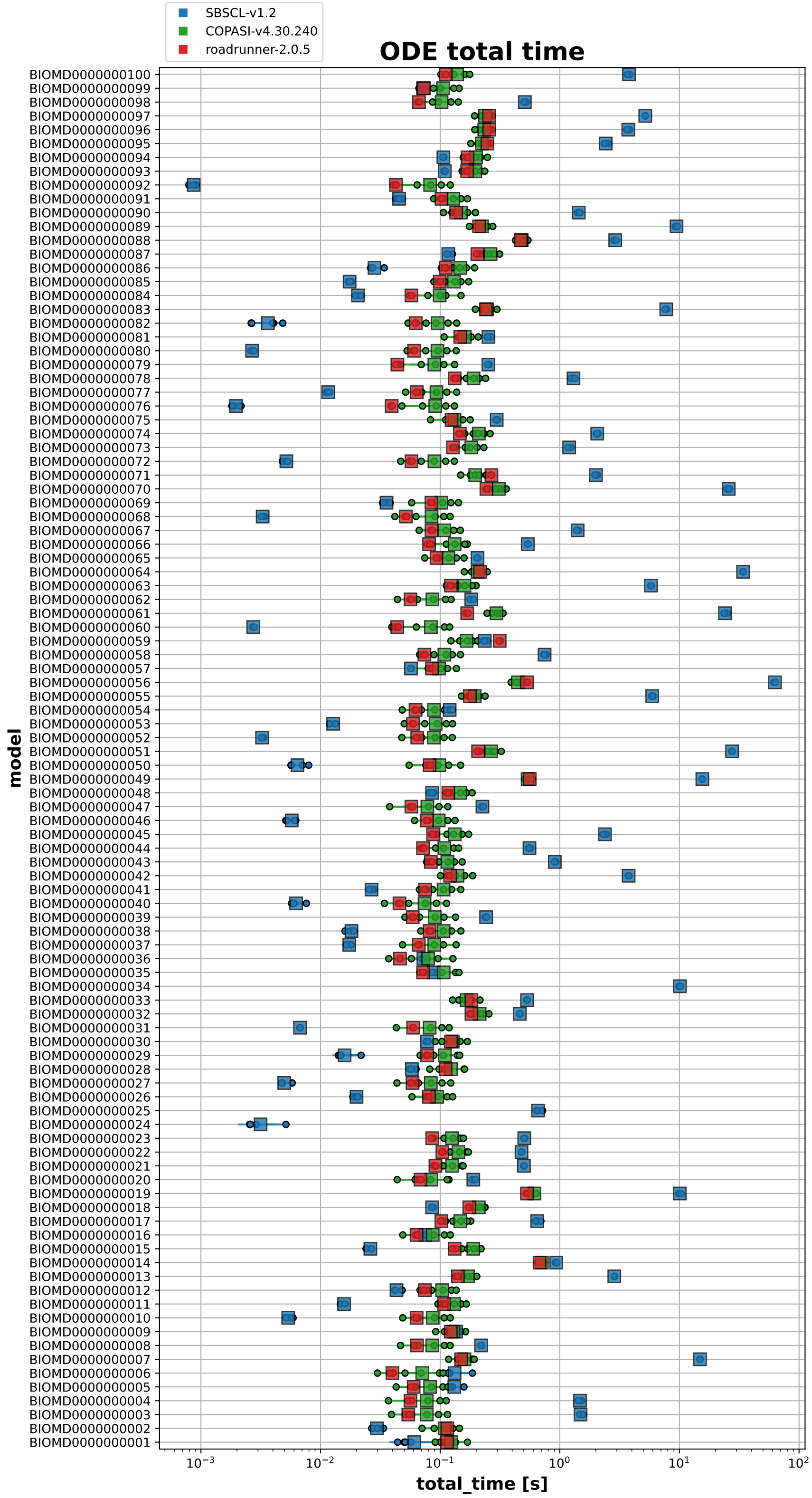

Figure 6. BioModels load and simulate time (in s). Displayed is mean and standard deviation for five repeats and the individual repeats for loading and simulating the first 100 models from BioModels Database[27] with SBSCL version 2.1, COPASI version 4.30.240[18], and RoadRunner version 2.0.5[40].

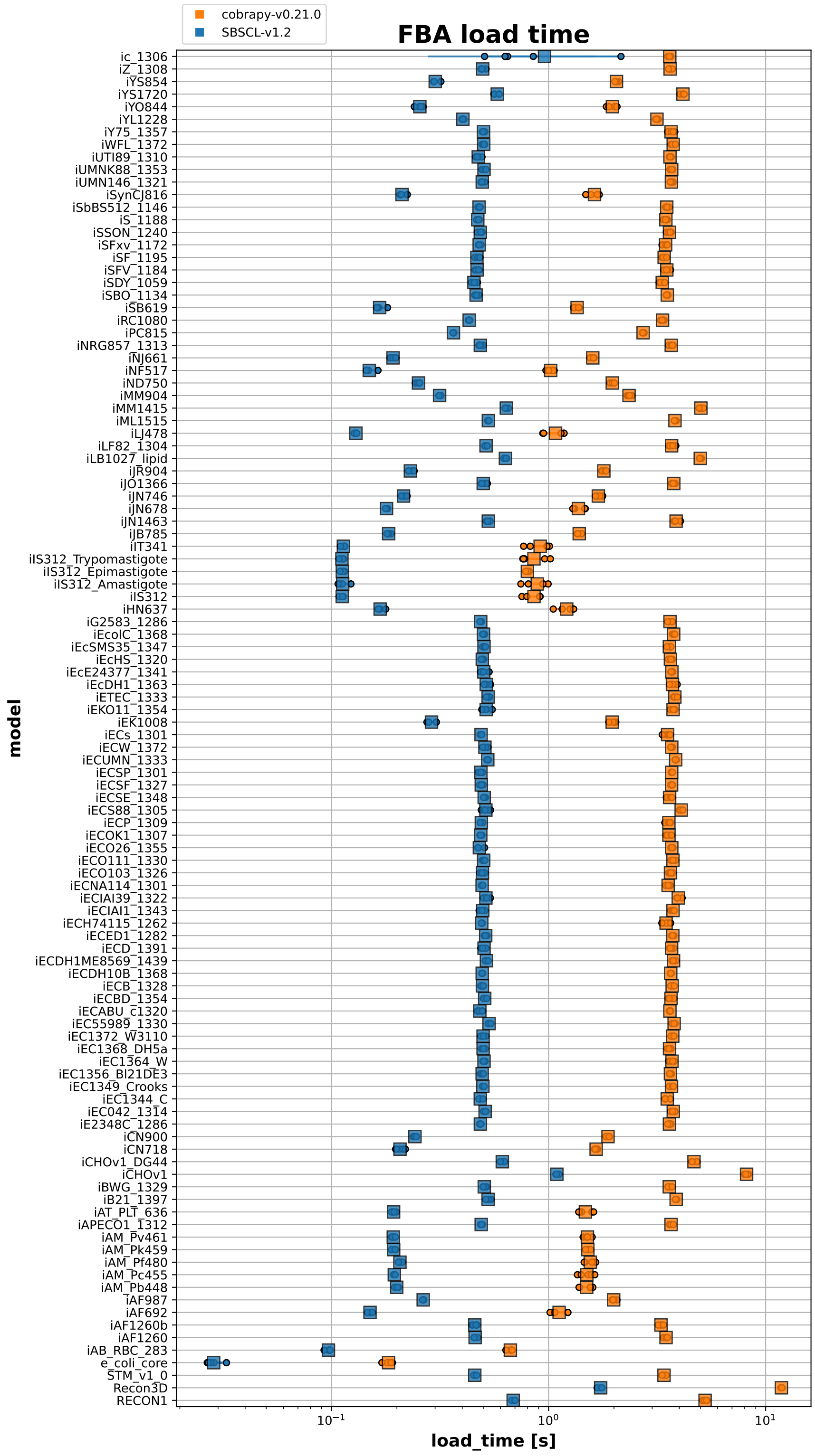

Figure 7. Biochemically, Genomically, Genetically structured (BiGG) Models load time (in s). Displayed is mean and standard deviation for five repeats and the individual repeats for loading all models from the BiGG Models Database[30] with SBSCL version 2.1 and COBRApy version 0.21.0[12].

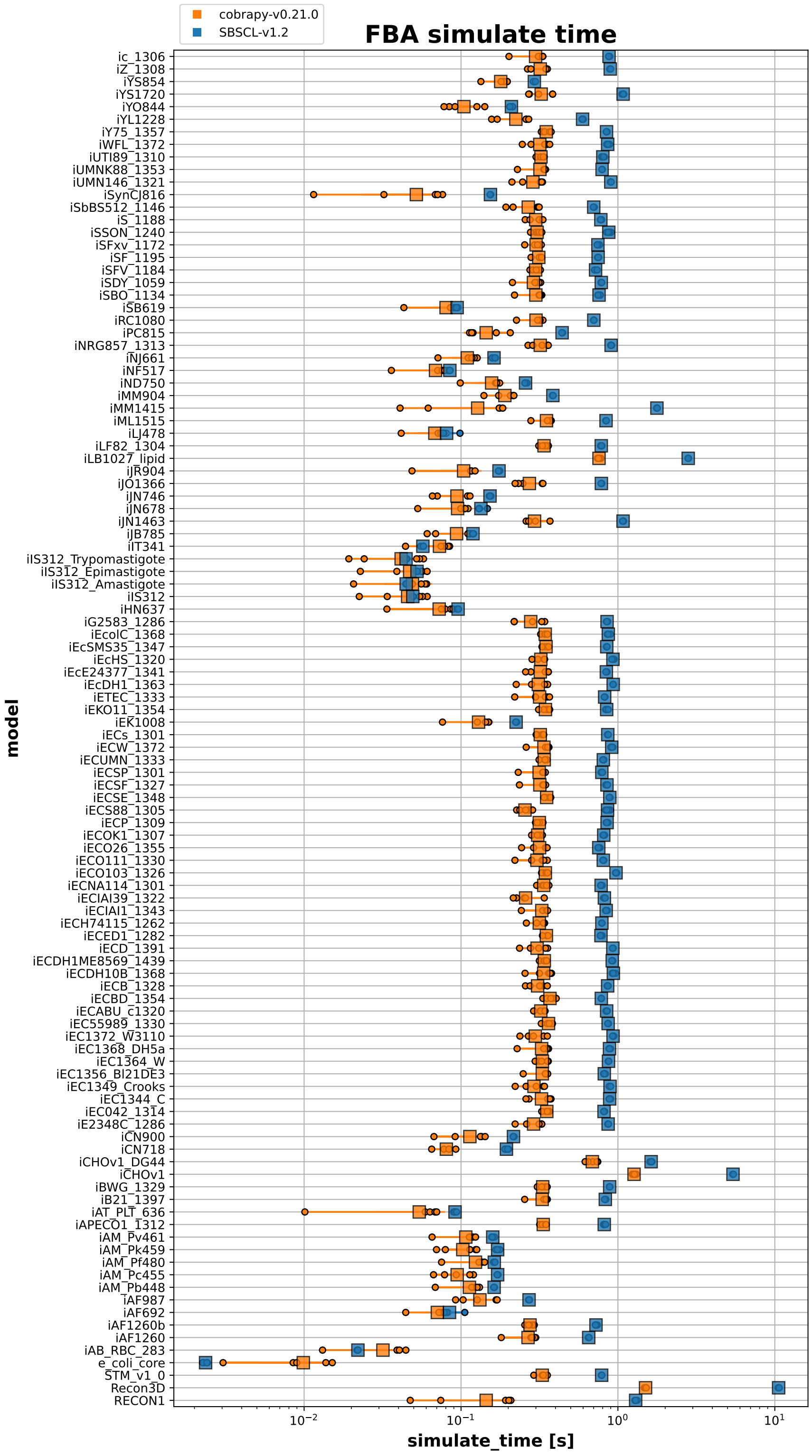

Figure 8. BiGG Models simulate time (in s). Displayed is mean and standard deviation for five repeats and the individual repeats for simulating all models from the BiGG Models Database[30] with SBSCL version 2.1 and COBRApy version 0.21.0[12].

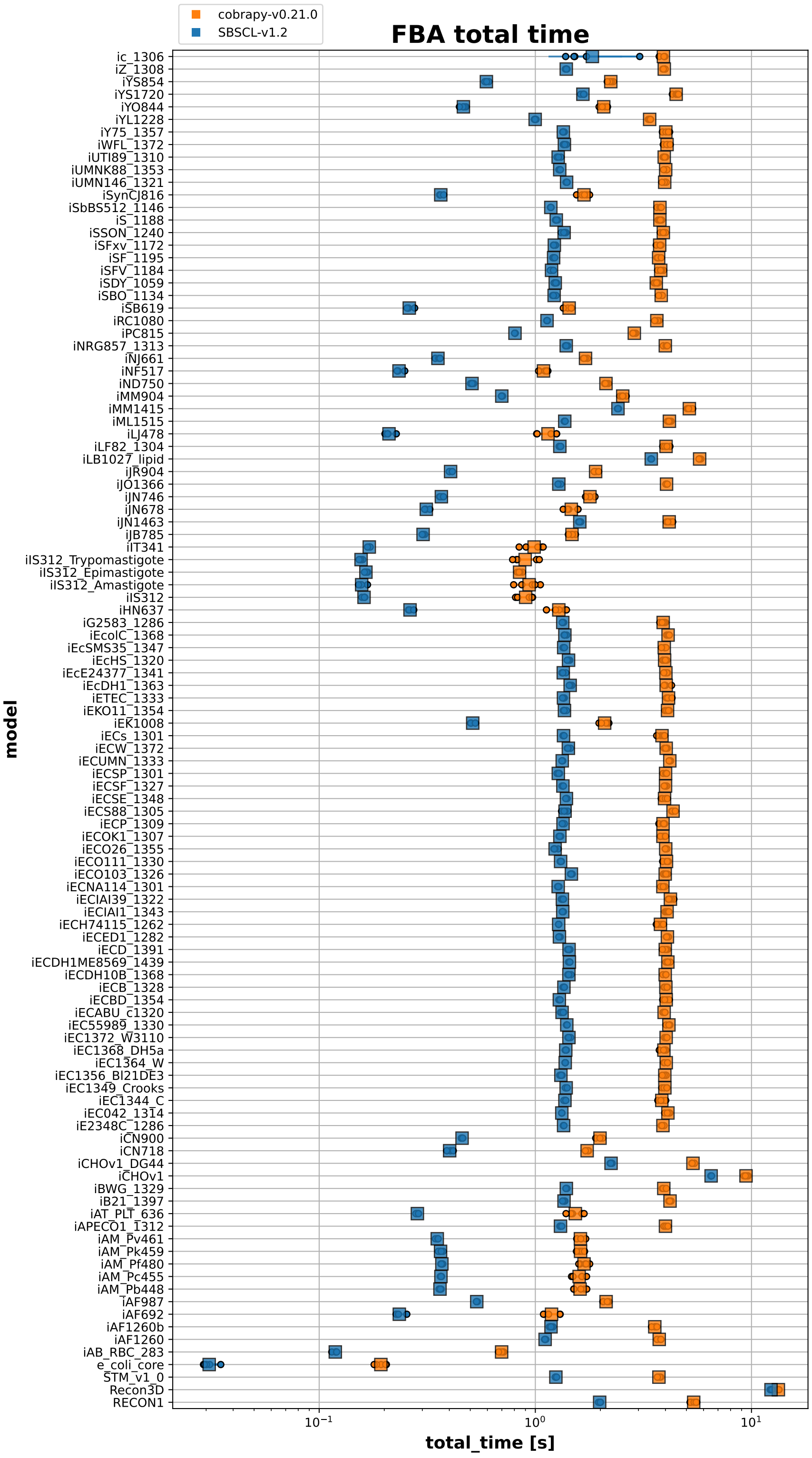

Figure 9. BiGG Models load and simulate time (in s). Displayed is mean and standard deviation for five repeats and the individual repeats for loading and simulating all models from the BiGG Models Database[30] with SBSCL version 2.1 and COBRApy version 0.21.0[12].

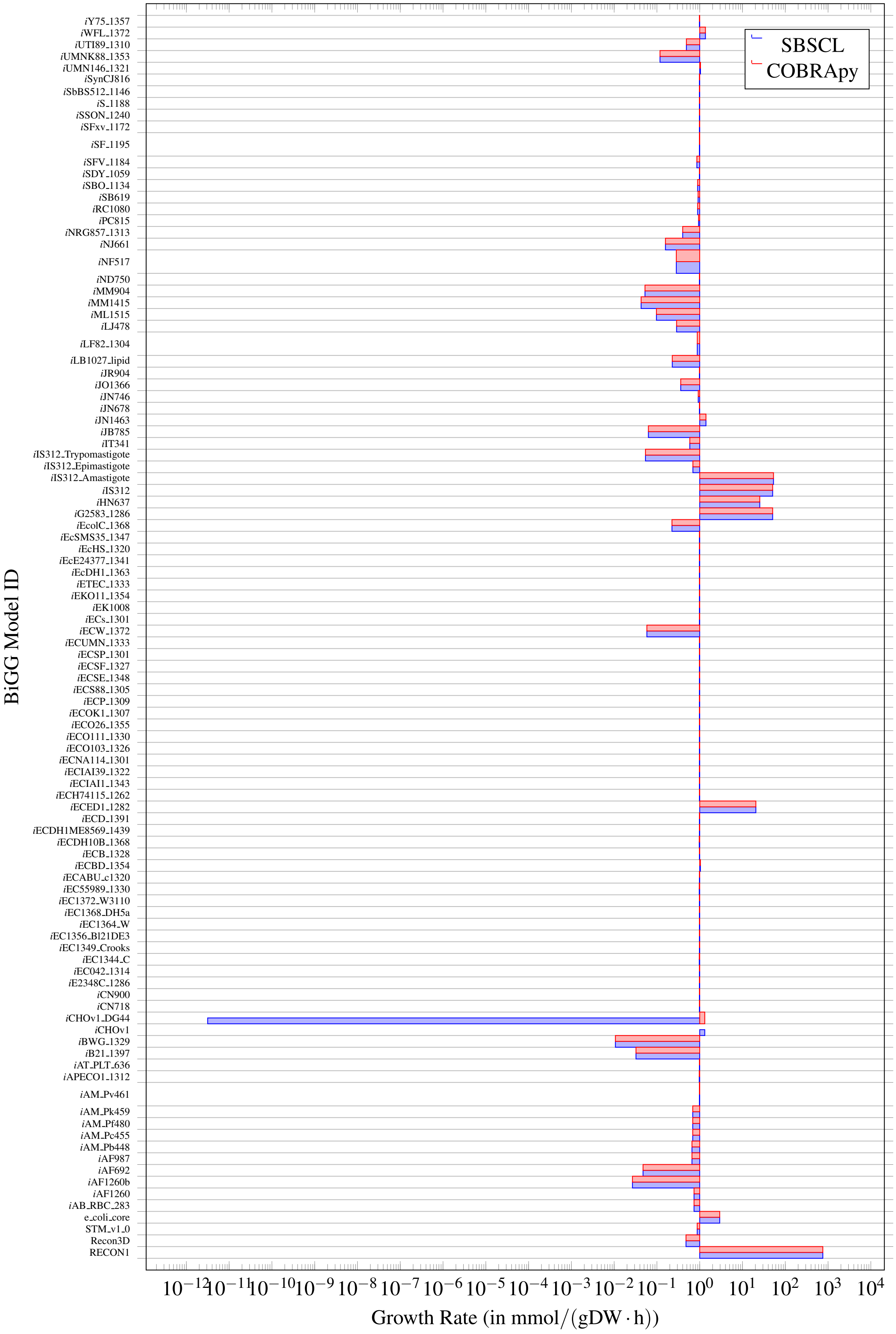

Figure 10. The difference in the objective values between SBSCL and COBRApy for all BiGG models.

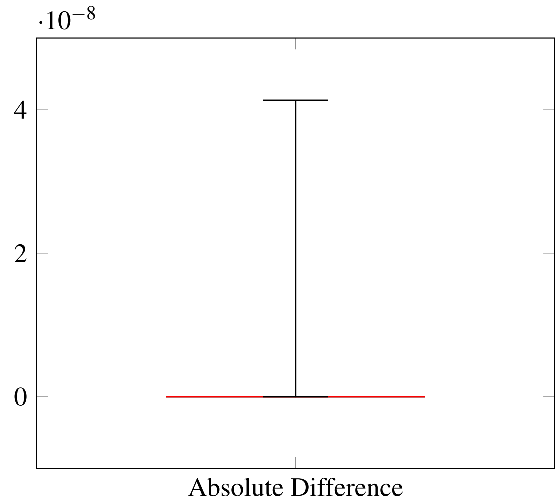

Figure 11. Divergence of the simulation results between SBSCL and COBRApy for all 108 models from BiGG.

3.4 Comparison to other simulators with SBML support

The SBML software guide lists several tools that solve and simulate SBML models in various frameworks. We selected all those tools from this list, for which a report on supported SBML test cases is provided in the SBML Test Suite Database (on April 23rd, 2021) and that are not superseded or replaced by a reimplementation (i.e., with a newer version of the same software). For example, libRoadRunner replaces the earlier software RoadRunner[40]. Table 1 on the following page presents an overview of all tools with their main features, which are subsequently described in more detail.

3.4.1 AMICI

The Advanced Multilanguage Interface for CVODES and IDAS (AMICI) supports differential equation models encoded in the formats PySB or SBML. It automatically compiles such models into executable formats for simulation. The supported output formats include Python modules, C++ libraries, or Matlab files in `.mex` format[14]. The compiled simulation files also facilitate forward sensitivity analysis, steady-state sensitivity analysis, and adjoint sensitivity analysis for likelihood-based output functions[19].

3.4.2 BioUML

BioUML is an integrated environment for systems biology and collaborative analysis of biomedical data[25]. It comes with the support of SBML[33], Systems Biology Graphical Notation (SBGN)[4,43], the Biological Pathway Exchange (BioPAX) format[7], PSI-MI, Open Biological and Biomedical Ontologies (OBO), and CellML[26] standards, along with providing different solvers for differential equations and methods for data analysis.

3.4.3 COPASI

COPASI is a standalone program designed for simulating and analysing biochemical networks and their dynamics[18]. This software application supports models in the SBML format (even with arbitrary discrete events). To simulate the behaviour of these models, COPASI provides implementations of ODEs solvers or Gillespie's stochastic simulation algorithm[17].

3.4.4 FluxBalance

This teaching tool for FBA supports the `fbc`[31,32] for SBML Level 3[20,21], including mapping flux distributions to SBML layouts[15]. Other simulation frameworks are not supported.

3.4.5 iBioSim

The computer-aided design (CAD) tool iBioSim aims to model, analyse, and design genetic circuits[46]. It primarily targets genetic circuits and analyses models representing metabolic networks, cell-signalling pathways, and other biological and chemical systems[28]. It supports importing all levels and versions of SBML and can export up to SBML Level 3 Version 1[21].

3.4.6 LibRoadRunner

LibRoadRunner is a high-performance and portable simulation engine for systems and synthetic biology[19,40]. It comes up with the support of ODE solver, code for structural analysis, steady-state analyses, event handling, stochastic simulation with a significant C and C++ Application Programming Interface (API), and Python bindings.

Table 1. Tool comparison

Software	Version	Operating Systems	Language	GUI	SBML Support	SBML Test Suite Database Results	SED-ML Support	Stochastic Tests
AMICI	0.11.16	Windows Linux macOS	Interfaces for C++, Python, MATLAB	☐	SBML import in Python and MATLAB interfaces	↗	☐	☐
BioUML		independent		☑	Supports SBML Level 1 Versions 1-2, Level 2 Versions 1-4, Level 3 Version 1	↗	☑	Passes DSMTS test suite
COPASI	4.30.240	Windows Linux macOS	C++ with multiple bindings	☑	Import and export of models from Level 1 to Level 3	↗		☑
FluxBalance	1.10	Windows	C#	☑	SBML with Layout and SBML extension package for Flux Balance Constraints (`fbc`) package in version 1 and 2	↗	☐	☐
iBioSim	3.0.0	Windows Linux macOS	Java™	☑	Imports all levels of SBML, exports up to L3V1. Support for fast reactions, `comp`, layout, `fbc`, and arrays	↗	☐	☑
libRoadRunner	2.0.1	Windows Linux macOS	C++, C with Python bindings	☐	SBML Level 2 to 3, excluding algebraic rules and delay differential equations	↗		
LibSBMLSim	1.4.0	independent	C	☐	SBML core	↗	☐	☐
modelbase	1.3.1	independent	Python		SBML core	↗	☐	☐
Morpheus	2.2.1	Windows Linux macOS	C++	☑	SBML core, `comp`	↗	☐	☐
SBSCL	2.1	independent	Java™	☐	All levels of SBML (core), `fbc` version 1 and 2, `comp` (through JSBML flattening)	↗	☑	☑
WinBEST-KIT	2.0.10	Windows	Closed source	☑	SBML core	↗	☐	☐

3.4.7 LibSBMLSim

LibSBMLSim provides a C implementation of SBML core with support for Ordinary Differential Equations (ODEs), Differential Algebraic Equations (DAEs), and Delay Differential Equations (DDEs) and bindings to many languages (including Java™, C#, Python, and Ruby)[19,42].

3.4.8 ModelBase

ModelBase is a free and expandable Python implementation for dynamic modelling, simulation, and analysis with SBML support[44].

3.4.9 Morpheus

Morpheus is an environment for modelling, simulation, and studying multi-scale as well as multicellular systems[41].

3.4.10 WinBEST-KIT

WinBEST-KIT is a simulator of biochemical reaction networks based on a GUI[36].

4 Known limitations

The project's issue tracker at `/draeger-lab/SBSCL/issues` is available online to request new features or report any problems and limitations of SBSCL.

Version 2.1 of SBSCL correctly solves the vast majority of the SBML Test Suite core test cases, as section 3.1 on page 8 indicates. A few test cases exist in which SBSCL currently fails to simulate correctly due to the following reasons:

- missing support for the delay property for the newly added `rateOf` function in SBML Level 3 Version 2 (see issue 46).
- some tests trigger events before the event condition is fulfilled (see issue 44).

In addition, some test cases have a stochastic nature which can fail sporadically. A compromise between runtime for the continuous integration and accuracy of the simulation results (depending on the number of runs used for averaging) had to be chosen.

Most failing test cases are related to the SBML extension package for the Hierarchical Model Composition (`comp`) package[37] for SBML Level 3. SBSCL uses the JSBML library[10,34] to flatten `comp` models and subsequently simulate the flattened models. Due to issues in JSBML with flattening the models, the `comp` package test cases fail to simulate. The issue is tracked via an issue in the JSBML repository at `/sbmlteam/jsbml/issues/213`. After resolving the flattening issue, the remaining `comp` tests will pass. Importantly, this is not a simulation issue with SBSCL, which supports all mathematical features in the `comp` model, but solely a problem of converting the hierarchical model to a non-hierarchical one.

All mentioned issues are tracked on the issue tracker and will be resolved in future versions of SBSCL.

Acknowledgements

The authors acknowledge contributions by Nicolas Rodriguez, Alexander Dörr, Roland Keller, Dieudonné M. Wouamba, Akito Tabira, Akira Funahashi, Michael J. Ziller, Richard Adams, Nicolas Rodriguez, Noriko Hiroi, and the Harvey Mudd College for the ODEToolkit.

Funding: The National Resource for Network Biology (NRNB) and Google Inc. supported this work as part of their summer of code programs (GSoC). AD was funded by the German Center for Infection Research (DZIF), grant № 8020708703, and supported by infrastructural funding from the *Deutsche Forschungsgemeinschaft* (DFG, German Research Foundation), Cluster of Excellence EXC 2124 Controlling Microbes to Fight Infections. MK is supported by the Federal Ministry of Education and Research (BMBF, Germany) within the research network Systems Medicine of the Liver (LiSyM, grant № 031L0054) and by the DFG within the Research Unit Programme FOR 5151 "QuaLiPerF (Quantifying Liver Perfusion–Function Relationship in Complex Resection—A Systems Medicine Approach)" by grant № 436883643.

Conflict of interest: None

Abbreviations

API Application Programming Interface
AMICI Advanced Multilanguage Interface for CVODES and IDAS
BiGG Biochemically, Genomically, Genetically structured
BioPAX Biological Pathway Exchange

**BMBF**	Federal Ministry of Education and Research
**CAD**	computer-aided design
**COMBINE**	Computational Modeling of Biological Networks
**comp**	SBML extension package for the Hierarchical Model Composition
**COPASI**	COmplex PAthway SImulator
**COBRA**	Constraint-Based Reconstruction and Analysis
**CSV**	Comma-Separated Values
**DAE**	Differential Algebraic Equation
**DDE**	Delay Differential Equation
**DFG**	*Deutsche Forschungsgemeinschaft*, German Research Foundation
**DZIF**	German Center for Infection Research
**FBA**	Flux Balance Analysis
**fbc**	SBML extension package for Flux Balance Constraints
**FERN**	Framework for Evaluation of Reaction Networks
**GLPK**	GNU Linear Programming Kit
**GSoC**	Google Summer of Code
**GUI**	Graphical User Interface
**NRNB**	National Resource for Network Biology
**OBO**	Open Biological and Biomedical Ontologies
**ODE**	Ordinary Differential Equation
**OMEX**	Open Modeling EXchange format
**POM**	Project Object Model
**SBGN**	Systems Biology Graphical Notation
**SBML**	Systems Biology Markup Language
**SBSCL**	Systems Biology Simulation Core Library
**SDE**	Stochastic Differential Equation
**SED-ML**	Simulation Experiment Description Markup Language
**UML**	Unified Modeling Language

References

1. Adams, R., Moraru, I. et al. jlibSEDML-a Java library for working with SED-ML. *Nature Precedings*, pp. 1–1, 2010.
2. Bergmann, F.T., Adams, R. et al. COMBINE archive and OMEX format: one file to share all information to reproduce a modeling project. *BMC Bioinformatics*, 15:369, December 2014 doi: 10.1186/s12859-014-0369-z.
3. Bergmann, F.T., Cooper, J. et al. Simulation experiment description markup language (sed-ml) level 1 version 2. *Journal of integrative bioinformatics*, 12:262, September 2015 doi: 10.2390/biecoll-jib-2015-262.
4. Bergmann, F.T., Czauderna, T. et al. Systems biology graphical notation markup language (SBGNML) version 0.3. *Journal of Integrative Bioinformatics*, 17(2-3):20200016, June 2020 doi: 10.1515/jib-2020-0016.
5. Bornstein, B.J., Keating, S.M. et al. LibSBML: an API Library for SBML. *Bioinformatics*, 24(6):880–881, March 2008 doi: 10.1093/bioinformatics/btn051.
6. Buchweitz, L.F., Yurkovich, J.T. et al. Visualizing metabolic network dynamics through time-series metabolomic data. *BMC Bioinformatics*, 21(1):130, April 2020 doi: 10.1186/s12859-020-3415-z.
7. Demir, E., Cary, M.P. et al. The BioPAX community standard for pathway data sharing. *Nature biotechnology*, 28(9):935–942, December 2010 doi: 10.1038/nbt.1666.
8. Dörr, A., Keller, R. et al. SBMLsimulator: a Java tool for model simulation and parameter estimation in systems biology. *Computation*, 2(4):246–257, December 2014 doi: 10.3390/computation2040246.
9. Dräger, A. and Palsson, B.Ø. Improving collaboration by standardization efforts in systems biology. *Frontiers in Bioengineering*, 2(61), December 2014 doi: 10.3389/fbioe.2014.00061.
10. Dräger, A., Rodriguez, N. et al. JSBML: a flexible Java library for working with SBML. *Bioinformatics*, 27(15):2167–2168, June 2011 doi: 10.1093/bioinformatics/btr361.
11. Dräger, A. and Waltemath, D. Overview: Standards for Modeling in Systems Medicine. In Wolkenhauer, O., editor, *Systems Medicine*, volume 3, pp. 345–353. Academic Press, Oxford, September 2020.

12. Ebrahim, A., Lerman, J.A. et al. COBRApy: COnstraints-Based Reconstruction and Analysis for Python. *BMC Systems Biology*, 7:74, August 2013 doi: 10.1186/1752-0509-7-74.
13. Erhard, F., Friedel, C.C. et al. FERN – a Java framework for stochastic simulation and evaluation of reaction networks. *BMC Bioinformatics*, 9:356, August 2008 doi: 10.1186/1471-2105-9-356.
14. Fröhlich, F., Weindl, D. et al. AMICI: High-Performance Sensitivity Analysis for Large Ordinary Differential Equation Models, 2020.
15. Gauges, R., Rost, U. et al. The Systems Biology Markup Language (SBML) Level 3 Package: Layout, Version 1 Core. *J. Integr. Bioinform.*, 12(2), 2015 doi: 10.2390/biecoll-jib-2015-267.
16. Gillespie, D.T. A General Method for Numerically Simulating the Stochastic Time Evolution of Coupled Chemical Reactions. *Journal of Computational Physics*, 22(4):403–434, December 1976 doi: 10.1016/0021-9991(76)90041-3.
17. Gillespie, D.T. Exact Stochastic Simulation of Coupled Chemical Reactions. *The Journal of Physical Chemistry*, 81(25):2340–2361, 1977 doi: 10.1021/j100540a008.
18. Hoops, S., Sahle, S. et al. COPASI–a COmplex PAthway SImulator. *Bioinformatics*, 22(24):3067–3074, December 2006 doi: 10.1093/bioinformatics/btl485.
19. Hucka, M., Bergmann, Frank T., D.A. et al. SBML Test Suite Database. `http://sbml.org/Facilities/Database/Simulator`, August 2021.
20. Hucka, M., Bergmann, F.T. et al. Systems Biology Markup Language (SBML) Level 3 Version 2 Core Release 2. *Journal of Integrative Bioinformatics*, 16(2):1, June 2019 doi: 10.1515/jib-2019-0021.
21. Hucka, M., Bergmann, F.T. et al. Systems Biology Markup Language (SBML) Level 3 Version 1 Core. *Journal of Integrative Bioinformatics*, 15(1):1, April 2018 doi: 10.1515/jib-2017-0080.
22. Hucka, M., Smith, L.P. et al. SBML Test Suite release 3.3.0. `https://doi.org/10.5281/zenodo.1112521`, December 2017.
23. Keller, R., Dörr, A. et al. The systems biology simulation core algorithm. *BMC Systems Biology*, 7:55, July 2013 doi: 10.1186/1752-0509-7-55.
24. King, Z.A., Lu, J.S. et al. BiGG Models: A platform for integrating, standardizing, and sharing genome-scale models. *Nucleic Acids Research*, October 2015 doi: 10.1093/nar/gkv1049.
25. Kolpakov, F., Akberdin, I. et al. BioUML: an integrated environment for systems biology and collaborative analysis of biomedical data. *Nucleic Acids Research*, 47(W1):W225–W233, May 2019 doi: 10.1093/nar/gkz440.
26. Lloyd, C.M., Halstead, M.D.B. et al. CellML: its future, present and past. *Prog Biophys Mol Bio*, 85(2-3):433–450, 2004 doi: 10.1016/j.pbiomolbio.2004.01.004.
27. Malik-Sheriff, R.S., Glont, M. et al. BioModels—15 years of sharing computational models in life science. *Nucleic Acids Research*, 48:D407–D415, January 2020 doi: 10.1093/nar/gkz1055.
28. Myers, C.J., Barker, N. et al. iBioSim: a tool for the analysis and design of genetic circuits. *Bioinformatics*, 25(21):2848–2849, November 2009 doi: 10.1093/bioinformatics/btp457.
29. Neal, M.L., König, M. et al. Harmonizing semantic annotations for computational models in biology. *Briefings in Bioinformatics*, 20(2):540—-550, November 2018 doi: 10.1093/bib/bby087.
30. Norsigian, C.J., Pusarla, N. et al. BiGG Models 2020: multi-strain genome-scale models and expansion across the phylogenetic tree. *Nucleic Acids Res.*, 11 2019 doi: 10.1093/nar/gkz1054, gkz1054.
31. Olivier, B.G. and Bergmann, F.T. The Systems Biology Markup Language (SBML) Level 3 Package: Flux Balance Constraints. *Journal of Integrative Bioinformatics*, 12:269, September 2015 doi: 10.2390/biecoll-jib-2015-269.
32. Olivier, B.G. and Bergmann, F.T. SBML Level 3 Package: Flux Balance Constraints version 2. *Journal of Integrative Bioinformatics*, 15, March 2018 doi: 10.1515/jib-2017-0082.
33. Renz, A., Mostolizadeh, R. et al. Clinical Applications of Metabolic Models in SBML Format. In Wolkenhauer, O., editor, *Systems Medicine*, volume 3, pp. 362–371. Academic Press, Oxford, January 2020.
34. Rodriguez, N., Thomas, A. et al. JSBML 1.0: providing a smorgasbord of options to encode systems biology models. *Bioinformatics*, June 2015 doi: 10.1093/bioinformatics/btv341.
35. Scheper, T., Klinkenberg, D. et al. A mathematical model for the intracellular circadian rhythm generator. *The Journal of neuroscience : the official journal of the Society for Neuroscience*, 19:40–47, January 1999.
36. Sekiguchi, T., Hamada, H. et al. WinBEST-KIT: Biochemical reaction simulator that can define and customize algebraic equations and events as GUI components. *Journal of bioinformatics and computational biology*, 17:1950036, December 2019 doi: 10.1142/S0219720019500367.
37. Smith, L.P., Hucka, M. et al. SBML Level 3 package: Hierarchical Model Composition, Version 1 Release 3. *Journal of Integrative Bioinformatics*, 12:268, September 2015 doi: 10.2390/biecoll-jib-2015-268.
38. Smolen, P., Baxter, D.A. et al. A reduced model clarifies the role of feedback loops and time delays in the *Drosophila* circadian oscillator. *Biophysical journal*, 83:2349–2359, November 2002 doi: 10.1016/S0006-3495(02)75249-1.

39. Smolen, P., Hardin, P.E. et al. Simulation of Drosophila circadian oscillations, mutations, and light responses by a model with VRI, PDP-1, and CLK. *Biophysical journal*, 86:2786–2802, May 2004 doi: 10.1016/S0006-3495(04)74332-5.

40. Somogyi, E.T., Bouteiller, J.M. et al. libRoadRunner: a high performance SBML simulation and analysis library. *Bioinformatics*, 31:3315–3321, October 2015 doi: 10.1093/bioinformatics/btv363.

41. Starruß, J., de Back, W. et al. Morpheus: a user-friendly modeling environment for multiscale and multicellular systems biology. *Bioinformatics*, 30:1331–1332, May 2014 doi: 10.1093/bioinformatics/btt772.

42. Takizawa, H., Nakamura, K. et al. LibSBMLSim: A reference implementation of fully functional SBML simulator. *Bioinformatics*, April 2013 doi: 10.1093/bioinformatics/btt157.

43. Touré, V., Dräger, A. et al. The Systems Biology Graphical Notation: Current Status and Applications in Systems Medicine. In Wolkenhauer, O., editor, *Systems Medicine*, volume 3, pp. 372–381. Academic Press, Oxford, January 2020.

44. van Aalst, M., Ebenhöh, O. et al. Constructing and analysing dynamic models with modelbase v1.2.3: a software update. *BMC bioinformatics*, 22:203, April 2021 doi: 10.1186/s12859-021-04122-7.

45. Waltemath, D., Adams, R. et al. Reproducible computational biology experiments with SED-ML-the simulation experiment description markup language. *BMC Systems Biology*, 5(1):1–10, 2011 doi: 10.1186/1752-0509-5-198.

46. Watanabe, L., Nguyen, T. et al. iBioSim 3: A Tool for Model-Based Genetic Circuit Design. *ACS Synthetic Biology*, 8(7):1560–1563, 2019 doi: 10.1021/acssynbio.8b00078, PMID: 29944839.

47. Yurkovich, J.T., J., Y.B. et al. A Padawan Programmer's Guide to Developing Software Libraries. *Cell Systems*, 5(5):P431–437, October 2017 doi: 10.1016/j.cels.2017.08.003.
